# Effects of a Home-Based Prehabilitation Program on Biomarkers and Hospital Outcomes in Total Knee Arthroplasty: A Protocol for a Randomized Controlled Trial

**DOI:** 10.7759/cureus.87061

**Published:** 2025-06-30

**Authors:** Gustavo Halmenschlager, Ana Leal, José Carlos Albarello, Yan Razuck, Thiago Lemos

**Affiliations:** 1 Laboratory of Neuromuscular Research and Exercise Physiology, National Institute of Traumatology and Orthopedics, Rio de Janeiro, BRA; 2 Teaching and Research Division, National Institute of Traumatology and Orthopedics, Rio de Janeiro, BRA

**Keywords:** arthritis, biological markers, exercise, knee osteoarthritis, rehabilitation

## Abstract

Background

Prehabilitation programs have gained attention for their potential to improve surgical outcomes in patients undergoing total knee arthroplasty (TKA). Exercise may modulate inflammatory markers (e.g., CRP and IL-6), nutritional and coagulation profiles, and joint-specific biomarkers, factors that influence postoperative recovery. However, the effects of preoperative exercise on these biological mechanisms in TKA patients remain insufficiently investigated.

Methods

This trial presents the protocol for an eight-week, semi-supervised, home-based prehabilitation program designed for patients scheduled for TKA. Participants will be randomized into an intervention group, which will perform exercises with remote supervision, or a control group, which will receive usual care. The primary outcomes included changes in inflammatory, nutritional, and coagulation biomarkers pre- and post-TKA. The secondary outcomes included hospital length of stay, postoperative complications, self-reported pain, stiffness, function, and satisfaction. Data will be collected at baseline, before surgery, and during postoperative follow-up.

Discussion

Home-based interventions may offer a scalable alternative to traditional prehabilitation, improving accessibility in large healthcare systems. By integrating structured preoperative exercise with remote supervision, this study sought to determine whether prehabilitation can modulate key biomarkers, enhance functional recovery, and reduce surgical risk. The results will contribute to the growing body of evidence supporting home-based prehabilitation strategies, providing insights into their potential role in optimizing TKA recovery. These findings may inform future rehabilitation protocols and healthcare policies, particularly in resource-limited public health systems.

The trial was registered with ClinicalTrials.gov under the identifier RBR-4HSKT2D on March 18, 2025.

## Introduction

Osteoarthritis is the most common form of arthritis, and its prevalence is projected to increase by 75% by 2050 owing to population aging [1]. Characterized by pain, stiffness, progressive inflammation, and degeneration of articular cartilage [2], osteoarthritis imposes a substantial global socioeconomic burden [1]. As the disease advances, many patients with knee osteoarthritis require total knee arthroplasty (TKA), which is a procedure with increasing demand [1].

Patients with osteoarthritis exhibit significantly lower physical activity levels than healthy older adults [3]. In this context, prolonged TKA waiting times exacerbate inactivity, contributing to muscle weakness, functional decline [4], increased comorbidities, and poorer recovery [5]. Importantly, low preoperative functional capacity is associated with poor recovery, increased surgical complications, prolonged hospital stay, and higher readmission rates, further straining healthcare systems [6].

To mitigate these issues, clinical guidelines recommend preoperative exercise programs to improve postoperative recovery. Prehabilitation enhances physical readiness for surgery, improving their ability to tolerate surgical stress, potentially improving pain management, functional capacity, and quality of life [7]. In this regard, home-based exercise programs offer a viable alternative to in-person rehabilitation by overcoming barriers such as financial constraints and limited access [8]. Notably, supervised home-based interventions demonstrate comparable efficacy to in-person rehabilitation in improving pain and function [9].

While research on TKA often emphasizes functional and pain-related outcomes, biomarkers also represent a critical area of investigation. They offer valuable insights into preoperative assessment, intraoperative response, risk of postoperative complications, rehabilitation effectiveness, and tissue regeneration [10]. Research has explored biomarker-based patient selection and outcome prediction as well as the effects of exercise on biomarker modulation and recovery. For instance, patients with osteoarthritis often exhibit elevated baseline levels of systemic inflammatory markers such as C-reactive protein (CRP) [11] and lactate [12]; and a locally disturbed balance of proinflammatory and anti-inflammatory cytokines, such as interleukin-6 (IL-6), IL-1β, α-tumor necrosis factor (TNF-α), and IL-10 [13]. However, exercise can mitigate inflammation, prevent cartilage degeneration, and reduce subchondral bone loss, while modulating biomarkers linked to postoperative recovery and survival [14]. Nonetheless, despite the feasibility and cost-effectiveness of supervised home-based prehabilitation, the relationship between this intervention, biomarker levels, and hospital outcomes remains unclear.

This study aims to evaluate the effects of a semi-supervised home-based prehabilitation program on systemic and joint-specific biomarkers, including CRP, IL-6, IGF-1, and irisin, in patients undergoing TKA. Additionally, it assesses the program’s impact on hospital length of stay, complication rates, readmissions, and self-reported pain, function, stiffness, and satisfaction. By addressing the current lack of standardized home-based prehabilitation protocols, this study may guide evidence-based implementation in public health settings, particularly where access to traditional in-person rehabilitation is limited.

## Materials and methods

Ethical approval and protocol registration

This study protocol was approved by the local ethics committee (approval number: 83209024.1.0000.5273) and registered on the national clinical trial registry (ReBEC) at “ensaiosclinicos.gov.br” under identifier RBR-4hskt2d. All study procedures adhered to the Consolidated Standards of Reporting Trials (CONSORT) guidelines. Participants in the study were required to sign a written informed consent form. However, participants were free to withdraw from the study at any time without any consequences. Informed consent was obtained individually, in a private setting, by the principal investigator and supervising physician after a detailed explanation of the study procedures. All experimental procedures were conducted in compliance with the ethical principles of the Declaration of Helsinki.

Trial design

**Figure 1:**
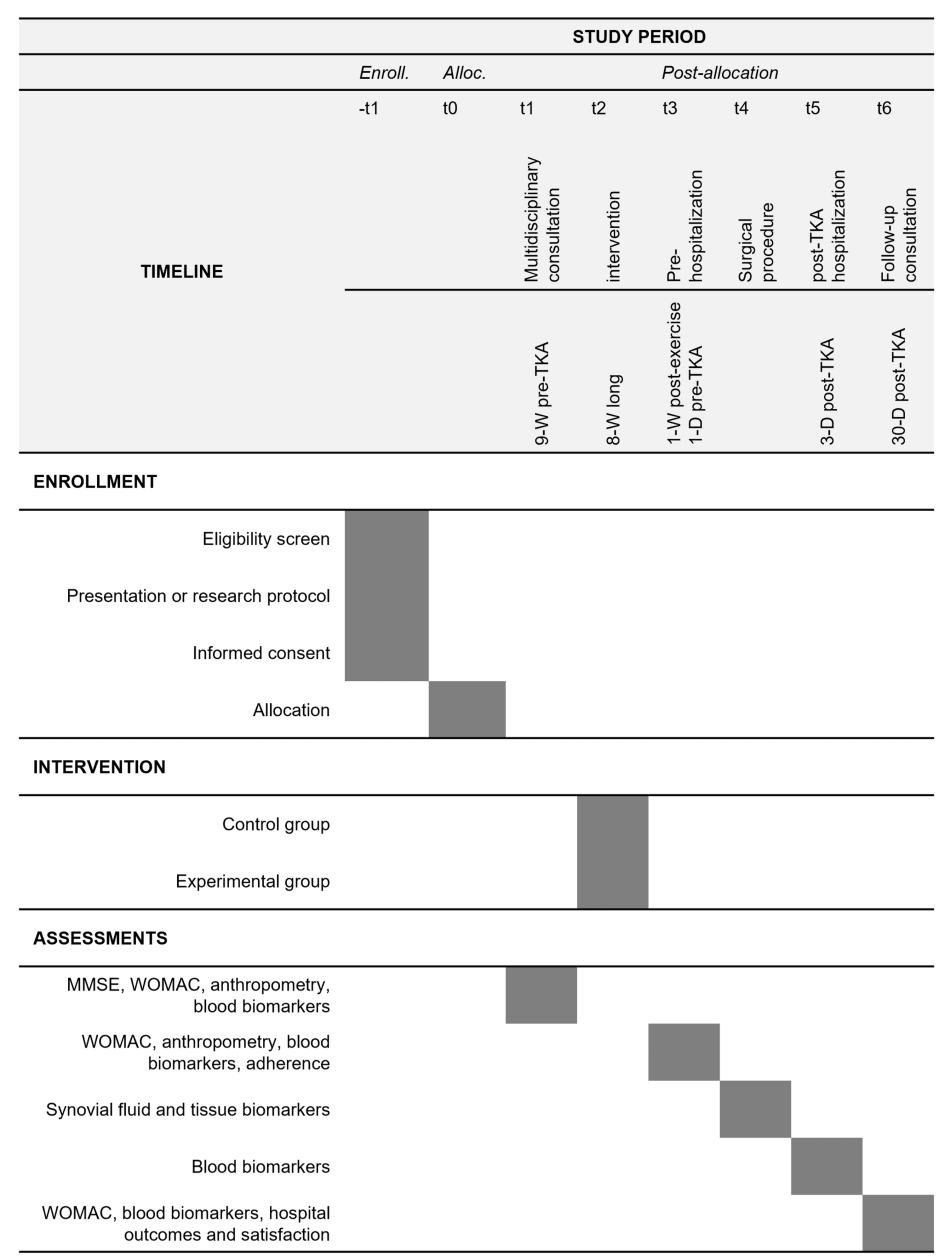
SPIRIT diagram of the study design and timeline. Illustration of the study schedule for enrollment, interventions, and assessments, based on the SPIRIT guidelines. Enroll., enrollment; Alloc., allocation; TKA, total knee arthroplasty; -W, weeks; -D, days; MMSE, Mini-Mental State Exam; WOMAC, Western Ontario and McMaster Universities Osteoarthritis Index; SPIRIT, Standard Protocol Items: Recommendations for Interventional Trials

**Figure 2:**
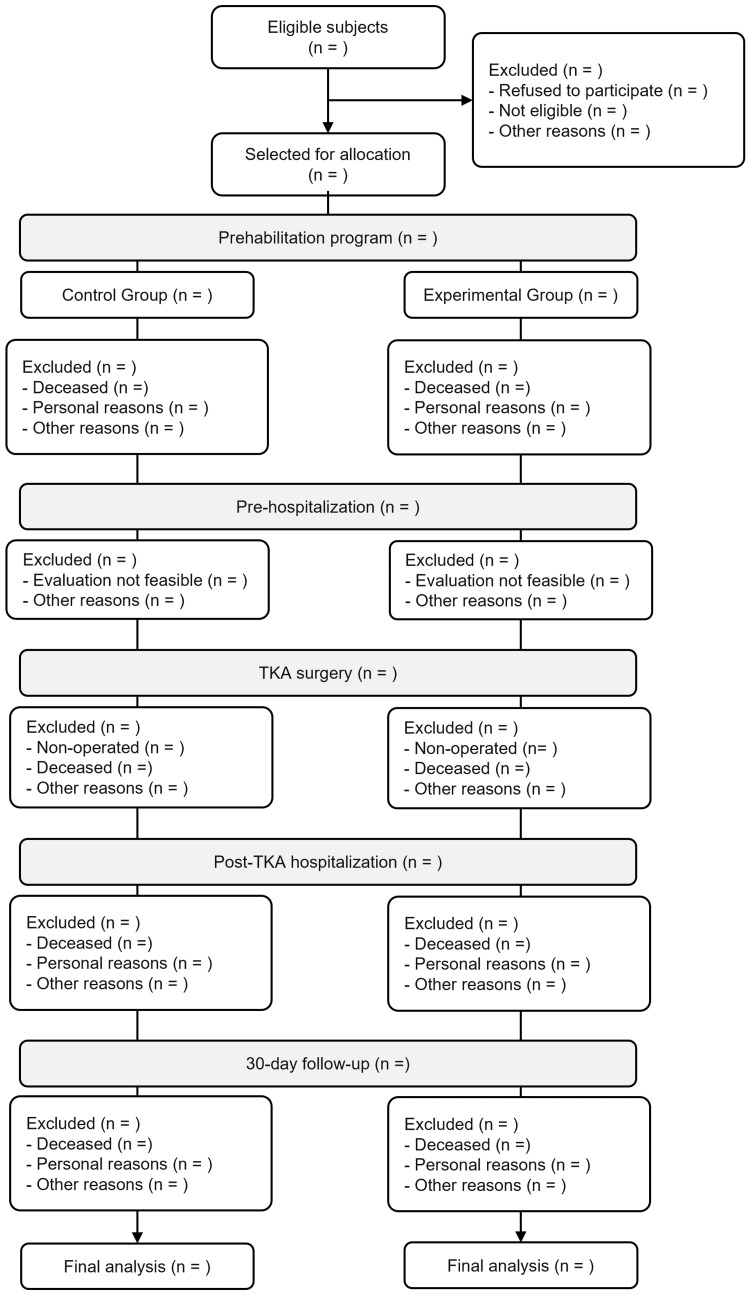
Participant flow chart throughout the study phases. This flow chart outlines the process of participant inclusion, allocation, and follow-up throughout the study. It displays the number of patients screened, randomized, excluded, and analyzed in both experimental and control groups across the various stages of the study.

This study is a single-center, two-arm, parallel-group randomized controlled trial conducted at a national orthopedic reference hospital. The interventional design was developed following the SPIRIT (Standard Protocol Items: Recommendations for Interventional Trials) guidelines (Figure 1). The recruitment timeline of this prospective trial will be contingent on available human resources, with an estimated 15-20 patients enrolled concurrently throughout the study period (Figure 2).

Participants and recruitment procedure

A convenience sample will be recruited from patients with advanced knee osteoarthritis scheduled for primary TKA at a national reference orthopedic hospital within the public healthcare system. Eligible participants will be approached in person during a multidisciplinary consultation nine weeks before surgery and invited to participate in the study. Before randomization, all participants will receive comprehensive information about the study, including the assessment procedures and the intervention protocol.

Enrollment

Men and women aged 50 to 75 years will be included if they are deemed capable of understanding and agreeing to the informed consent terms and are not currently engaged in structured exercise programs at least once per week or have not performed regular exercise for at least three months before the study. This age range was selected based on institutional epidemiological data, as the majority of TKA candidates treated at our center fall within this demographic. Eligible patients must have a diagnosis of knee osteoarthritis based on the Ahlbäck classification and be scheduled for TKA by their physician. Participants or family members must have access to WhatsApp interactions, as well as availability for telerehabilitation sessions. Only those without clinical or orthopedic conditions contraindicating regular physical exercise, as determined by clinical and physiotherapy evaluation, will be included.

Patients will be excluded if they fail to attend scheduled telerehabilitation sessions or respond to WhatsApp communication. Those experiencing unexpected medical complications, such as infections or cardiac or respiratory events, will be instructed to discontinue the program and withdraw from the study. The exclusion criteria also included changes in clinical status leading to surgical contraindications or new injuries during the prehabilitation period that prevented participation in home exercises. Additionally, participants with cognitive deficits were excluded based on the Portuguese version of the Mini-Mental State Examination (MMSE), with cut-off scores of ≤20 for illiterate individuals, ≤25 for primary school level, ≤26.5 for junior high school, ≤28 for high school, and ≤29 for higher education [15].

Randomization

After the explanatory meeting, eligible participants who complete baseline assessments will be randomized using simple randomization with a 1:1 allocation ratio via Randomizer® (www.randomizer.org). The allocation sequence will be generated by an independent researcher not involved in enrollment or assessment. Allocation concealment will be ensured using sequentially numbered, opaque, sealed envelopes, which will be opened only after confirming participant eligibility.

Control group

The participants in the control group will receive an institutional educational booklet containing standard preoperative physiotherapy recommendations. The only exercise instructions before TKA provided in the booklet were as follows: “(1) Move your feet up and down 20 times (active movement while lying on a treatment table, plantar dorsiflexion) and (2) inhale deeply through your nose while raising your arms and exhale through your mouth as you lower them. Perform 20 repetitions.” Participants in the control group will only follow these institutional procedures and will not engage in a home-based program. They will be instructed to maintain their usual daily activities and adhere to the recommendations provided in the booklet.

Experimental group

Patients in the experimental group will receive the institutional booklet and participate in a semi-supervised home-based prehabilitation program. Before starting, they will undergo theoretical and practical instruction to ensure proper exercise execution, as outlined in Table 1. Additional time will be allocated during the initial phase to overcome individual digital barriers, and all participants in the intervention group will receive in-person training on how to access video calls, send messages, and interact with the WhatsApp platform. The research team will verify movement accuracy, provide guidance to family members for additional support, and monitor any changes in the participants' physical or mental conditions. The training protocol was designed for a home setting without the need for specialized equipment and was adapted from the tele-prehabilitation model proposed by Guida et al. [16]. It consists of (1) warm-up, (2) balance exercises, and (3) muscle strengthening exercises. Sessions will be conducted three times per week over eight weeks, with at least 48 hours of rest between sessions, totaling a maximum of 24 training sessions.

**Table 1:** Home-based exercise protocol. *Estimated time.

	Exercise	Sets	Repetitions	Rest	Time*
Warm-up					
	Seated knee flexion and extension	3	10 movements per leg	No rest	2 min
	Marching in place	3	30 movements	30 s	4 min
Balance					
	Single-leg stance	3	15 s per side	30 s	3 min
	Tandem walking (heel-to-toe)	3	10 steps in each direction	30 s	5 min
Strength					
	Sit-to-stand	3	10	30 s	5 min
	Wall calf raises	3	10	30 s	5 min

To enhance adherence and technique, participants will receive a link to an instructional video (Video 1) and a training diary to log their weekly exercise frequency (Table 2 of Appendices). Additionally, weekly videoconference sessions provide real-time feedback, while WhatsApp monitoring facilitates communication, allowing participants to ask questions via text, images, audio, or video messages. WhatsApp was selected because of its accessibility and ease of use. To further minimize attrition, the research team will send daily reminder messages via WhatsApp each morning, reinforcing engagement. Weekly live video calls will also serve to optimize exercise execution and encourage adherence.

**Video 1:** Instructional video demonstrating proper exercise techniques for patients in the home-based prehabilitation program.

Primary outcome

In TKA, the extent of surgical trauma significantly influences postoperative pain and recovery. Plasma CRP, a well-established biomarker of tissue injury and inflammation, is commonly used to monitor recovery due to its rapid response, high specificity for acute inflammation, and ease of measurement, especially in elderly arthroplasty patients. CRP was selected as the primary outcome because of its association with osteoarthritis severity, surgical response, and postoperative complications, typically peaking on the third postoperative day [17].

Blood samples will be collected in an outpatient setting at four periods: before and after the eight-week prehabilitation program and three and 30 days post-TKA in both the experimental and control groups. The preoperative measurement one day before surgery (t3 in Figure 1) was timed to reflect chronic systemic adaptations resulting from prehabilitation while minimizing the confounding influence of acute exercise responses. This decision is supported by evidence showing that CRP typically returns to baseline (or a new post-exercise baseline) within six days after cessation of exercise [18]. The third postoperative day (t5) was selected to capture the peak inflammatory response following surgery, as supported by TKA-specific literature [17]. This time point also coincides with the typical hospitalization period, facilitating consistent, in-hospital sample collection. In the long term, given the anti-inflammatory effects of physical exercise, sustained reductions in CRP levels may reflect the effectiveness of the intervention in modulating systemic inflammation and promoting recovery [14].

Given that CRP levels can be influenced by factors such as sleep deprivation and acute infections, additional control measures will be implemented. Participants will report their average sleep duration and quality during the week preceding each blood draw (except for the third-day post-TKA measurement), as insufficient or disturbed sleep is associated with elevated CRP levels. Additionally, clinical symptoms suggestive of viral or bacterial infections (e.g., fever, sore throat, cough, or malaise) will be assessed before each collection since such conditions may increase CRP levels and confound the interpretation of the intervention's effects [19]. These precautions aimed to strengthen the internal validity of CRP as a primary outcome by minimizing interference from unrelated acute inflammatory processes.

Additional blood biomarkers

Beyond CRP, additional biomarkers will be assessed simultaneously to strengthen inflammatory indicators and provide insights into tissue regeneration, nutritional status, and thromboembolic risks associated with TKA and exercise. After collection, part of the blood samples will be immediately used for assessment of hematological and lipid profile parameters (complete blood count and lipidogram), albumin, prealbumin, lactate, and coagulation system factors (fibrinogen, D-dimer, plasminogen activator inhibitor-1 (PAI-1), and tissue plasminogen activator). The other part of the sample will be centrifuged to obtain plasma that will be stored at -80°C and used in the future for measurement of insulin-like growth factor-1 (IGF-1), IL-1β, and IL-6. Additionally, the myokines irisin and lubricin will be evaluated. IL-6 was chosen for its role in cytokine regulation and its sensitivity to both surgical trauma and exercise [14,20]. IGF-1 reflects anabolic and regenerative processes, supporting muscle recovery in aging populations [14,21]. Exercise induces the release of myokines such as irisin and lubricin, which may modulate pain and delay osteoarthritis progression via mitochondrial metabolic reprogramming [22], while lactate may indicate inadequate perfusion [23]. Irisin was included due to its association with metabolic modulation and potential chondroprotective effects, especially relevant in osteoarthritis progression and exercise adaptation [22].

Synovial fluid biomarkers

Synovial fluid will be collected immediately before the surgical incision, following sterile field placement and ischemia, using a 20 mL syringe with a 1.2 × 40 mm needle. The sample will be centrifuged at 500 × g for 5 min, and the supernatant will be aliquoted and stored at -80°C until analysis. Cytokine levels (IL-1β, IL-4, IL-12, IL-6, IL-8, IL-10, IL-1β, and TNF-α) will be assessed using a flow cytometry assay (BD™ Cytometric Bead Array (CBA) Human Inflammatory Cytokine Kit). Free DNA levels will be measured using a DNA intercalating probe (Picogreen).

Tissue biomarkers

Osteoarthritis progression involves subchondral bone alterations, which exercise may counteract by preserving cartilage and bone integrity [14,20]. Bone cartilage fragments (distal femur condyle and tibial plateau) will be collected post-osteotomy, stored in sterile tubes, and processed for mitochondrial activity assays and immunohistochemical analysis of extracellular matrix structure. Markers include fibronectin type III domain-containing 5 (FNDC5), β-galactosidase (senescence marker), Bcl-2-associated X protein (BAX), and cytochrome C (Cyt-C). These markers will provide insights into mitochondrial function, programmed cell death, and cartilage remodeling, helping evaluate the local physiological response to the exercise program. Mitochondrial membrane permeability will be assessed using flow cytometry (Mitochondrial Membrane Potential Kit, MAK160, Merck). Due to considerable inter-individual variability and limited evidence directly connecting these biomarkers to prehabilitation in OA, FNDC5/irisin and β-galactosidase levels should be interpreted as exploratory outcomes. Effect sizes are expected to be small to moderate. Nevertheless, FNDC5 has demonstrated chondroprotective mechanisms in OA models, including enhanced cartilage integrity and reduced chondrocyte apoptosis [24], supporting its inclusion to explore metabolic and cellular adaptations to exercise.

Self-reported pain, stiffness, knee function, and anthropometric measurement

The Western Ontario and McMaster Universities Arthritis Index (WOMAC), a validated tool for knee osteoarthritis and post-TKA patients, assesses pain (5 items, 0-20 points), stiffness (2 items, 0-8 points), and physical function (17 items, 0-68 points) [25]. The total score is obtained by summing the individual scores and can be converted to a 0-100 scale, with higher values indicating greater impairment. Body mass will be measured using the Líder® P150C digital scale (São Paulo, Brazil). Height will be assessed using a Ghrum Polar Manufacture Instruments® anthropometer (Geneva, Switzerland). Waist circumference will be measured with Sanny® anthropometric tape (São Paulo, Brazil). Both the WOMAC and the anthropometric measurements will be performed at three moments: multidisciplinary consultation, pre-hospitalization, and 30-day follow-up consultation.

Prehabilitation adherence

Adherence to the prehabilitation program will be assessed by calculating the percentage of completed sets compared to the total prescribed sets. This information will be recorded in the training diary and reported by the patient or a family member. The number and classification of adverse events occurring during the home-based training period will be recorded separately from hospital outcomes.

Adherence will be operationally defined as the completion of ≥75% of prescribed sessions, based on adherence rates commonly reported in the literature. A recent systematic review by Adebero et al. [7] found that most prehabilitation trials achieved adherence rates of 74% or higher, supporting the use of this threshold. This cutoff will be used to determine eligibility for per-protocol analyses evaluating intervention efficacy, while all randomized participants will be retained in the dose-response analysis, regardless of adherence level.

Hospital outcomes

Hospital outcomes will include the length of hospital stay (in days), hospital readmission rate, and number and classification of complications occurring within 30 days post-surgery. These indicators were selected to complement the biological and functional recovery metrics, offering system-level proxies for early recovery quality. Specifically, reduced length of stay may reflect improved physiological readiness for discharge, while lower readmission and complication rates may signal enhanced resilience and postoperative recovery. These data will be retrieved from patients’ medical records, which will be accessed using the registration number recorded during patient enrollment. Surgical complications will be defined as any adverse events based on the Clavien-Dindo classification [26].

Satisfaction level

Patient satisfaction will be assessed 30 days post-TKA using a validated TKA satisfaction questionnaire [27]. This time point was selected to capture the short-term functional and symptomatic benefits of prehabilitation when effects on recovery are most tangible. Four primary questions, rated on a five-point Likert scale, will evaluate pain relief, functional improvement, and overall satisfaction. Scores ranged from 0 (very dissatisfied) to 100 (very satisfied), with the total score calculated as the mean of all responses. The total satisfaction score will be obtained by summing the individual scores of the four satisfaction questions and dividing by four. If any individual component is missing, the total score will not be calculated.

Sample size

The sample size was estimated using repeated measures analysis of variance with four time points (nine weeks pre-TKA, one day pre-TKA, three days post-TKA, and 30 days post-TKA) and two groups (experimental and control). Assuming a moderate effect size (f = 0.25), α = 0.05, and 80% power (β = 0.20), 78 participants were required (G*Power 3.1.9.7, University of Kiel, Germany). This effect size was selected based on evidence from a study in TKA patients with CRP as the primary outcome, which reported a between-group difference of 9.12 mg/L and a pooled standard deviation of 10.95, corresponding to a Cohen’s d of 0.83 (f ≈ 0.29), and statistical power of 88% with 61 participants. Therefore, f = 0.25 was adopted as a conservative and evidence-based estimate for the current trial [28]. Accounting for a 10% potential attrition rate, the final target sample size was 86 participants.

Blinding

This study follows a single-blind design, in which only the data analyst remains blinded to group allocation. Due to the nature of the intervention (an exercise program), blinding participants or intervention providers was not feasible, as doing so would compromise adherence, safety, and protocol fidelity. Consequently, participants, data collectors, and outcome assessors will be aware of group assignments.

## Results

The significance level was set at p < 0.05, and analyses will be performed using IBM SPSS Statistics 21.0 (IBM®, Armonk, NY, USA). Data normality will be assessed using the Shapiro-Wilk test. Baseline demographic characteristics will be compared between the groups using independent t-tests (normal data), Mann-Whitney U tests (non-normal data), and chi-square or Fisher’s exact tests (categorical variables).

The primary outcome, changes in plasma CRP levels across four time points (nine weeks pre-TKA, one day pre-TKA, three days post-TKA, and 30 days post-TKA), and WOMAC scores across three time points (baseline, one day pre-TKA, and 30 days post-TKA) will be analyzed using two-way repeated-measures ANOVA (group × time interaction), contingent upon assumptions of normality and sphericity. If these assumptions are violated, linear mixed models (LMM) or generalized estimating equations (GEE) will be employed as more robust alternatives, capable of handling missing data and irregular intervals. Post-hoc pairwise comparisons will use the Bonferroni correction to adjust for type I errors. For non-parametric repeated measures, the Friedman test will be used, followed by Dunn-Bonferroni post-hoc tests when applicable.

Secondary outcomes, including synovial fluid and tissue biomarkers, hospital outcomes (length of stay, complications, readmissions), and satisfaction scores at specific time points, will be compared between groups using independent t-tests (normal data) or Mann-Whitney U tests (non-normal data). Correlations between biomarkers, hospital outcomes, WOMAC scores, and satisfaction will be examined using repeated measures correlation (rmcorr) for normal data or generalized linear mixed model (GLMM) for non-normal data. Rmcorr will be applied when assessing the strength and direction of associations between two continuous variables measured repeatedly within individuals. GLMM will be used in cases requiring the modeling of random effects, adjustment for covariates, or handling of non-normal or categorical outcomes, ensuring analytic flexibility across diverse data structures. To account for multiple comparisons in biomarker and exploratory analyses, false discovery rate (FDR) adjustment will be applied where appropriate.

Effect sizes (e.g., partial eta-squared for ANOVA and Cohen’s d for pairwise tests) and 95% confidence intervals will be reported alongside p-values to enhance the clinical interpretability of findings.

Missing data will be addressed using linear regression models to examine the dose-response relationship, including participants who did not reach the ≥75% adherence threshold as well as those who withdrew from the intervention due to comorbidities or frailty. These individuals will be retained in the analysis to reflect real-world variability.

## Discussion

The primary objective of this trial is to compare the effects of an eight-week semi-supervised home-based prehabilitation program with a control group on biomarkers in patients undergoing TKA. Secondary objectives include evaluating hospital outcomes (length of stay, complications, and readmission rates) and self-reported measures such as function, pain, stiffness, and satisfaction with surgery. We hypothesize that prehabilitation will lead to better outcomes, as prior studies show that exercises improve preoperative health, enhancing surgical recovery.

While postoperative rehabilitation is well-established, patients often undergo surgery in a debilitated state owing to sedentary lifestyles, which correlates with higher complication rates and worse clinical outcomes [6]. Home-based exercise programs are effective in increasing adherence among older adults and, when implemented preoperatively, can improve function, reduce pain and stiffness, lower surgical anxiety and incidence of complications, shorten hospitalization, and improve TKA satisfaction [7].

The day before surgery and the early postoperative period are critical phases of both surgical preparation and recovery. Preoperatively, biomarkers provide key insights into systemic inflammation, nutritional status, and thromboembolic risk, aiding surgical risk stratification and complication prediction. For instance, elevated preoperative CRP levels are an independent predictor of postoperative infection in arthroplasty patients [29]. Increased CRP and IL-6 levels indicate chronic inflammation, while reduced albumin and prealbumin levels suggest poor nutritional status, potentially impairing wound healing [30]. In the early postoperative phase, biomarkers reflect tissue injury, inflammation, and the progression of recovery. CRP levels peak around the third day post-surgery, indicating the magnitude of surgical trauma [17], while IL-6 levels rise earlier, marking the acute inflammatory response [20]. Lactate levels can reveal metabolic stress and inadequate perfusion [23], while IGF-1 plays a critical role in muscle regeneration [21]. Additionally, monitoring D-dimer and fibrinogen levels is crucial for assessing thromboembolic risk [31], while the presence of myokines, such as irisin and lubricin, suggests metabolic and joint-protective adaptations [22]. Finally, hemoglobin and hematocrit levels assist in identifying anemia, and coagulation markers [32] help assess thromboembolic risk.

Osteoarthritis is characterized by progressive cartilage degeneration and inflammation [2], and exercise mitigates these effects by preserving cartilage, reducing inflammation, and maintaining subchondral bone integrity [14]. Moreover, exercise influences the biomarkers linked to surgical recovery, promotes tissue regeneration, and optimizes inflammatory and thromboembolic responses. Physical activity stimulates growth factors, such as IGF-1, enhancing muscle repair and cartilage homeostasis, while mechanical loading regulates lubricin, which is crucial for joint lubrication [22]. Exercise also reduces systemic inflammation by lowering CRP and IL-6 levels [14] and preserving albumin and prealbumin levels, which are vital for wound healing and immune function. Additionally, it improves vascular function and fibrinolytic balance, thereby reducing the risk of thromboembolism.

Although biomarkers provide valuable insights into preoperative assessment, surgical response, postoperative complications, and tissue regeneration, the impact of prehabilitation on these markers in TKA patients has not been systematically investigated. Additionally, while home-based exercise programs may offer a feasible strategy to address healthcare system demands, the current lack of standardized protocols limits their structured implementation. Clarifying these protocols through rigorous studies such as this one is an important step toward refining perioperative care approaches and potentially enhancing surgical outcomes.

Strengths and limitations

We recognized potential challenges related to adherence and digital proficiency within our target age population (50-75 years). Cognitive impairments may also influence participation. However, we anticipate that video-based interactions will enhance motivation, accountability, and social support by closely replicating in-person coaching. Evidence suggests that frequent participant contact via telephone, Internet, or personal visits improves adherence to home-based exercise programs among older adults [33]. Telecommunication difficulties between participants and researchers are expected; however, to mitigate these issues, we will provide clear instructions for video call setup and allocate sufficient time to address technical challenges within our study schedule. We anticipate that technical barriers will be most prominent at the beginning of the program and diminish as participants become more accustomed to the process. Notably, a recent Brazilian study demonstrated that WhatsApp-based audio and visual messaging was effective in delivering a six-week psychosocial intervention to older adults, even without direct support from health professionals [34].

Another methodological limitation is the single-blind design, which may introduce performance and detection bias, particularly in subjective outcomes. To mitigate these biases, all outcome assessments will follow standardized protocols and be conducted by the same team of trained evaluators across both groups. Furthermore, participants in both arms will receive uniform instructions regarding their routine daily activities between baseline and surgery, minimizing behavioral discrepancies apart from the intervention itself. While self-reported outcomes are inherently prone to bias, they are measured using validated instruments with low response bias. In addition, we will perform sensitivity analyses to explore the robustness of results by accounting for potential confounders such as adherence, engagement levels, and expectancy effects. Importantly, the trial also includes objective outcomes, such as biomarkers, length of stay, complications, and anthropometric data, which enhance the overall internal validity of the findings despite the inherent limitations of a non-blinded behavioral intervention.

## Conclusions

To the best of our knowledge, this is the first protocol for a two-arm, single-center randomized controlled trial comparing the effects of a home-based prehabilitation program versus a control condition on biomarkers and hospital outcomes in patients undergoing total TKA. By incorporating preoperative exercises with remote supervision, this approach aims to optimize biomarker profiles before and after surgery, enhance patient satisfaction, and improve self-reported pain, stiffness, and knee function, while potentially reducing hospital stay and postoperative complications. The findings from this trial will contribute to the growing evidence base for home-based prehabilitation and may inform future rehabilitation strategies and public healthcare policies aimed at improving TKA outcomes, particularly in overburdened systems with long surgical waitlists.

## Appendices

**Table 2:** Training diary for patients to log weekly exercise frequency. The training diary will be given to patients at the baseline visit.

Workout 1					Date: __/__/____	
Exercise	Set 1	Set 2	Set 3	Difficulty		
Seated knee flexion and extension	( )	( )	( )	Easy ( )	Moderate ( )	Difficult ( )
Marching in place	( )	( )	( )	Easy ( )	Moderate ( )	Difficult ( )
Single-leg stance	( )	( )	( )	Easy ( )	Moderate ( )	Difficult ( )
Tandem walking (heel-to-toe)	( )	( )	( )	Easy ( )	Moderate ( )	Difficult ( )
Sit-to-stand	( )	( )	( )	Easy ( )	Moderate ( )	Difficult ( )
Wall calf raises	( )	( )	( )	Easy ( )	Moderate ( )	Difficult ( )
